# Microbial Community Restructuring and Functional Response in Giant Duckweed (*Spirodela polyrhiza*) Fronds Driven by Cadmium Stress

**DOI:** 10.3390/microorganisms13112423

**Published:** 2025-10-22

**Authors:** Bingliang Liu, Chen Yang, Xin Wan, Suming Chen, Yang Tao, Qiang Li, Hai Zhao, Xinhui Wang

**Affiliations:** 1 College of Food and Biological Engineering, Chengdu University, Chengdu 610106, China; liubingliang@cdu.edu.cn (B.L.); yangtao023@outlook.com (Y.T.);; 2 Institute for Advanced Study, Chengdu University, Chengdu 610106, China; 3 Agricultural Microbial Agents Key Laboratory of Sichuan Province, National Engineering and Research Center for Natural Medicines, Chengdu Institute of Biology, Chinese Academy of Sciences, Chengdu 610213, China

**Keywords:** cadmium, giant duckweed, phytoremediation, microbial response, microbial function

## Abstract

As a typical heavy metal pollutant, cadmium (Cd) poses significant threats to ecosystems and human health. Giant duckweed (*Spirodela polyrhiza*), a small aquatic plant characterized by rapid growth and efficient heavy metal accumulation, holds great promise for phytoremediation. However, the mechanisms by which *S. polyrhiza* enriches Cd—particularly the contributions of its surface-associated microbiota—remain poorly understood. In this study, *S. polyrhiza* fronds were exposed to 0, 1, and 10 μM Cd, and we observed a concentration-dependent increase in the abundance of epiphytic microorganisms on the frond surfaces. High-throughput 16S rRNA gene sequencing revealed that Cd stress significantly altered the diversity of the frond-epiphytic bacterial community. Notably, the relative abundances of the genera *Herbaspirillum*, *Enterobacter*, and *Pantoea* increased significantly with rising Cd concentrations. Functional prediction using PICRUSt2 indicated enrichment under Cd stress of specific traits—such as the nitrate/nitrite transporter NarK, signal transduction mechanisms, and ion channel proteins—suggesting these taxa may actively participate in Cd uptake and tolerance. Together, our results reveal a synergistic *S. polyrhiza*–microbiome response to Cd and identify taxa/functions as targets and biomarkers for microbe-augmented remediation.

## 1. Introduction

In recent decades, the rapid pace of industrialization has markedly exacerbated heavy metal contamination, particularly by cadmium (Cd), mercury (Hg), and lead (Pb) [1]. Among these, cadmium pollution is especially pervasive, owing to its widespread distribution in aquatic and terrestrial environments and its classification as one of the most serious global ecological threats [2]. The high water solubility and, chemical stability of cadmium, a persistent environmental pollutant, enable widespread dispersal and high bioavailability, driving substantial bioaccumulation and biomagnification along the food chain [3,4]. Such accumulation arrests growth and can even cause mortality in both fauna and flora [5,6]. In plants, cadmium toxicity exerts detrimental effects by inhibiting carbon fixation, reducing water content, decreasing biomass, diminishing chlorophyll levels, and impairing photosynthetic activity [7]. Cd can bind to proteins, leading to their denaturation and functional disruption [8]. Furthermore, cadmium compromises the plant’s capacity to uptake zinc and iron, thereby resulting in deficiencies of these essential nutrients [9]. At elevated concentrations, it suppresses plant growth and induces necrosis [10]. For example, in tomato, cadmium stress compromises cell-wall rigidity and alters turgor loss points, exacerbating leaf cell injury, impairing rehydration capacity, and increasing the risk of hydraulic failure [11]. In human populations, cadmium toxicity impacts multiple organs. Its primary accumulation occurs in the kidneys, resulting in severe damage including emphysema, renal tubular injury, and kidney stones [12]. Cadmium has a high bioaccumulation capacity in the body, notably in bones, where it causes substantial bone demineralization, leading to conditions such as osteoporosis and osteomalacia [13]. Inhalation exposure can induce chronic respiratory diseases and elevates the risk of lung cancer [14]. Mechanistically, cadmium has been shown to induce Leydig cell damage via activation of the TNF-α/TNFR1 signaling cascade and ROS-mediated necroptosis pathways [13]. Thus, cultivation of staple crops and vegetables in cadmium-tainted water poses a significant public health hazard [15,16]. Given these risks, the development of cost-effective and environmentally benign methods for cadmium removal is imperative for safeguarding both ecosystem integrity and human well-being.

Currently, a variety of physical, chemical, and biological approaches have been developed to remove Cd from water. Physical and chemical remediation methods include isolation, immobilization, electroremediation, and chemical precipitation, among others [17,18]. However, these approaches are often associated with limitations, such as high costs, intensive labor requirements, and the potential for causing secondary pollution [19]. In contrast, strategies that utilize plants and microorganisms to adsorb and transform pollutants are gaining increasing attention. Among these, phytoremediation is recognized as a viable in situ biological method for addressing heavy metal contamination. It is praised for its low infrastructure demands, minimal environmental impact, and capacity to preserve soil fertility and biodiversity [20,21]. A variety of plant types, including oil seed crops, grasses, and trees, have demonstrated the ability to absorb, stabilize, or degrade pollutants under controlled conditions [22]. Duckweed, a small free-floating aquatic plant, thrives across temperate and tropical water bodies—including lakes, ponds, and rice paddies—and tolerates a wide range of environmental conditions (2–35 °C, pH 3.5–10.5, and salinity 154–2276 mg/L) [23,24]. Its rapid growth rate, high biomass production, and robust heavy metal accumulation capacity have positioned duckweed as a promising candidate for phytoremediation applications [25]. For example, co-cultivation of duckweed with rice has been shown to reduce grain cadmium levels from 0.40 mg/kg to below 0.20 mg/kg, through mechanisms including soil-available Cd uptake, pH elevation, enrichment of Cd-immobilizing bacteria, and shifts in soil nitrogen speciation (increased ammonium and decreased nitrate) [24]. Despite these advances, the precise mechanisms by which duckweed efficiently accumulates cadmium remain largely unexplored.

The microbial community is increasingly recognized as a critical determinant of plant heavy metal tolerance. For example, soil-borne genera such as *Bacillus* sp. and *Burkholderia* sp. can facilitate Cd transport within the soil–root continuum [26]. An expanding body of evidence indicates that plants actively recruit beneficial microbial consortia to mount adaptive responses against heavy metal stress [27]. Plant-associated microbes synergistically influence Cd uptake through multiple mechanisms, including decreasing metal bioavailability, stimulating plant antioxidant defenses, modulating phytohormone levels via organic acid secretion, and directly mediating metal ion transport [28,29]. Moreover, recent work has demonstrated that plant growth-promoting rhizobacteria can activate rhizosphere nitrogen cycling, trigger specific root exudation profiles, and thereby recruiting synergistic microbial consortia; the establishment of such synthetic communities has been shown to enhance cadmium accumulation in the hyperaccumulator *Solanum nigrum* [30]. Similarly, cadmium stress has been reported to reshape the duckweed rhizosphere microbiome, with enrichments of Proteobacteria and Firmicutes potentially linked to heavy metal detoxification pathways [31]. Research has demonstrated that a two-step, plant-microbial combined remediation system, constituted by *S. polyrhiza* and a specific bacterial consortium, effectively removes Chemical Oxygen Demand (COD), color, and heavy metals (e.g., Cd, Ni) from textile wastewater [32]. Indeed, various bacterial taxa—including *Acinetobacter*, *Citrobacter*, *Bacillus*, *Curtobacterium*, *Frigoribacterium*, *Enterobacter*, *Methylobacterium*, *Erwinia*, and *Pantoea*—have been detected on duckweed surfaces [33]. Nevertheless, the specific microbial response mechanisms that underpin Cd adsorption and tolerance in duckweed remain poorly understood.

Therefore, the primary objectives of our study are as follows: (1) To characterize the physiological changes in giant duckweed (*Spirodela polyrhiza*) fronds under Cd stress, confirming the direct impact of Cd on the host plant. (2) To analyze Cd-induced structural shifts in the frond epiphytic (surface-associated) bacterial community. (3) To predict and interpret the functional adaptations of the microbial community. *S. polyrhiza* fronds were exposed to 0, 1, and 10 μM Cd treatments to assess microbial and structural responses. Scanning electron microscopy revealed a concentration-dependent increase in microbial colonization on frond surfaces under Cd stress. To characterize these shifts, we performed high-throughput 16S rRNA gene sequencing to analyze bacterial community diversity, composition, and predicted function. This study not only deepens our understanding of the adaptive strategies of giant duckweed to heavy metal stress but also provides novel insights for designing plant-microbe consortia to remediate Cd-contaminated aquatic environments.

## 2. Materials and Methods

### 2.1. Materials and Processing Conditions

Giant duckweed (*Spirodela polyrrhiza*) was collected in March 2025 from a rice field in Shuangliu District, Chengdu City, Sichuan Province, China, and maintained in the Chengdu University laboratory under accession number 001. *S. polyrhiza* was transferred to round plastic containers (20 L) containing site water and pre-cultured under constant temperature conditions of 25 ± 1 °C, a photoperiod of 16 h light/8 h dark, and a light intensity of 40 μmol m^−2^ s^−1^ to obtain healthy second-generation *S. polyrhiza*. Approximately 10 g of *S. polyrhiza*, with fronds measuring 6–8 mm in length, 5–6 mm in width, exhibiting green coloration and no necrosis, were selected and transplanted into 1000 mL beakers containing 800 mL of site water. To mimic the field matrix while holding aqueous chemistry constant across treatments, all exposures used a single, homogenized batch of field-collected surface water from the same rice field. Cadmium concentrations are reported as nominal (1 and 10 µM, added as CdCl_2_) because water chemistry (e.g., pH, hardness, DOC) can alter Cd speciation and bioavailability [34]; therefore, our inferences are restricted to comparative, dose-dependent responses within the same water matrix, not to absolute bioavailable Cd levels. The experiment comprised three Cd treatments—0, 1.0, and 10.0 µM Cd^2+^—applied for seven days, with three independent biological replicates per treatment (total: 90 g fresh mass across 9 beakers). Moreover, duckweeds typically double biomass every ~2–3 days under laboratory conditions, so a 7-day window spans multiple doublings and is sufficient to capture dose-dependent physiological and epiphytic-microbiome responses without artifacts from overgrowth. Cultivation conditions during exposure matched those of the pre-culture phase, and all treatments used the same water batch to minimize among-treatment variation in background chemistry.

### 2.2. Phenotypic Observation and Scanning Electron Microscopy

*S. polyrhiza* fronds exposed to 0, 1.0, and 10.0 μM Cd^2+^ for 7 days (designated CK, Cd1, and Cd10) were fixed with 2.5% (*v*/*v*) glutaraldehyde and maintained at 4 °C for 2–4 h. Following fixation, the samples were rinsed three times with 0.1 M potassium phosphate buffer and subsequently dehydrated through a graded ethanol series. Each frond was then mounted on a conductive stub, sputter-coated with gold, and imaged at 5 kV on a scanning electron microscope [19]. ImageJ software v1.8.0 quantified stomatal length, width, and pore area, and stomatal density (number per mm^2^) was calculated.

### 2.3. DNA Extraction and PCR Amplification

Intact fronds were gently rinsed to remove loosely suspended cells and processed without surface sterilization, so the 16S profiles predominantly reflect epiphytic communities, while a minor endophytic contribution cannot be fully excluded. For each treatment (CK, Cd1, Cd10), three biological replicates of *S. polyrhiza* frond tissue were processed using the TIANGEN DP302-02 genomic DNA kit according to the manufacturer’s instructions. DNA concentrations were determined by Qubit fluorometry (Invitrogen, Waltham, MA, USA). The V3–V4 region of the bacterial 16S rRNA gene was amplified using primers 341F (5′-CCTACGGGNGGCWGCAG-3′) and 805R (5′-GACTACHVGGGTATCTAATCC-3′) [35]. Each 25 µL PCR reaction contained 12.5 µL Phusion Hot Start Flex 2X Master Mix, 2.5 µL of each primer (10 µM), 50 ng template DNA, and nuclease-free water. Thermal cycling: 98 °C for 30 s; 32 cycles of 98 °C for 10 s, 54 °C for 30 s, 72 °C for 45 s; final extension at 72 °C for 10 min. Amplicons were purified with AMPure XT beads (Beckman Coulter, Brea, CA, USA) and quantified by Qubit.

### 2.4. Library Preparation, Sequencing, and Data Processing

Purified PCR products were assessed on an Agilent 2100 Bioanalyzer (Agilent Santa Clara, CA, USA) and quantified with the Illumina Library Quantitation Kit (Kapa Biosciences, Woburn, MA, USA). Libraries passing QC were sequenced (2 × 250 bp) on a NovaSeq 6000 platform (LC-Bio Technology, Hangzhou, China). Raw paired-end reads were demultiplexed by barcode, trimmed of adapters and primers with Cutadapt v1.9, merged with FLASH v1.2.8, and quality-filtered (Q < 20, length < 100 bp, ambiguous bases > 5%) using Fqtrim v0.94. Chimeric sequences were removed with Vsearch v2.3.4, yielding high-quality clean reads.

### 2.5. ASV Clustering and Taxonomic Annotation

High-quality clean reads were processed in QIIME2 v2021.4.0 using the DADA2 v2021.8.0 plugin for paired-end denoising, length filtering, and chimera removal [36]. This pipeline yielded representative amplicon sequence variants (ASVs) and their corresponding abundance table; singleton ASVs (those appearing only once across all samples) were excluded. Taxonomic assignment of each ASV was performed by aligning sequences against the SILVA v138 and NT-16S v20230718 databases, generating a comprehensive profile of bacterial taxa and their relative abundances at each taxonomic rank for subsequent phylogenetic and compositional analyses.

### 2.6. Diversity Analysis

Using the ASV abundance table and representative sequences, we assessed within-sample (α) and between-sample (β) diversity. α-Diversity metrics—observed ASVs, Chao1, and ACE (community richness), Shannon and Simpson indices (community diversity and evenness), and Good’s coverage (sequencing completeness)—were calculated in QIIME2 [37] and visualized with R (v4.1.3). For β-diversity, weighted UniFrac distance matrices quantified phylogenetic dissimilarities among samples; these distances were then ordinated via principal coordinate analysis (PCoA) in QIIME2 to reveal treatment-driven community shifts. Differentially abundant taxa were identified using Linear Discriminant Analysis Effect Size (LEfSe), with an LDA score threshold of 4, to pinpoint key microbial biomarkers associated with cadmium stress.

### 2.7. Functional Prediction

To predict the metabolic potential of the frond microbiome, we employed PICRUSt2 [38] to infer metagenomic content from 16S data. Predicted gene families were mapped to the Cluster of Orthologous Groups (COG) and Kyoto Encyclopedia of Genes and Genomes (KEGG) databases, enabling identification of functional pathways—such as transport systems, signal transduction, and ion-channel proteins—that are enriched or depleted under different Cd treatments.

### 2.8. Statistical Analysis

All experiments included three biological replicates per treatment. Quantitative measurements (stomatal dimensions and densities, diversity indices, and predicted functional abundances) were compared by one-way ANOVA followed by Tukey’s HSD test (*p* < 0.05) in SPSS 19.0. Data visualization and figure preparation were performed in Origin 2019 and Microsoft Excel.

## 3. Results

### 3.1. Growth Status of S. polyrhiza Fronds Under Different Cadmium Concentrations

**Figure 1:**
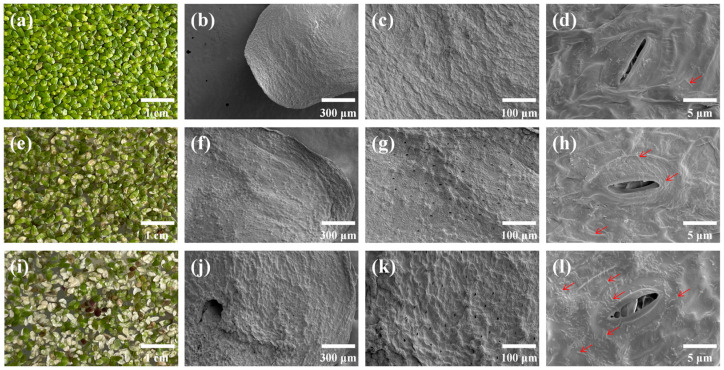
Growth status of *S. polyrhiza* under cadmium (Cd) stress at different concentrations. (**a**,**e**,**i**) Growth phenotypes after 7 days of treatment with 0, 1, and 10 μM Cd, respectively. (**b**–**d**) Scanning electron micrographs of Control (0 μM Cd) fronds. (**f**–**h**) Scanning electron micrographs of 1 μM Cd treatment fronds. (**j**–**l**) Scanning electron micrographs of 10 μM Cd treatment fronds. Red arrows indicate microorganisms adhered to the frond surface.

We used 1 and 10 µM Cd (~112 and ~1124 µg L^−1^) to span sub-lethal to inhibitory exposures for *S. polyrhiza* (7-day Lemna EC_50_ ≈ 1.9–24 µM; EC_10_ ≈ 1.5 µM [39]. In the control (CK), *S. polyrhiza* fronds remained turgid with uniform green pigmentation (Figure 1a). Exposure to 1 μM Cd (Cd1) caused mild chlorosis on a subset of fronds (Figure 1e), whereas 10 μM Cd (Cd10) led to extensive discoloration and visible wilting (Figure 1i). Our SEM analysis revealed pronounced, concentration-dependent changes in stomatal architecture on *S. polyrhiza* fronds (Figure 1, Table 1). After 7 days of Cd exposure, stomatal length did not differ among treatments (*p* > 0.05). By contrast, stomatal width and pore area increased significantly in Cd1 and Cd10, by 2.02–2.70-fold and 2.04–2.46-fold, respectively, relative to CK (*p* < 0.05). The largest mean width (3.38 μm) and area (29.81 μm^2^) occurred in Cd1, while Cd10 exhibited the highest stomatal density (189.11 mm^−2^). Notably, stomatal densities in Cd1 and Cd10 were 3.30× and 4.21× those of CK, respectively (*p* < 0.05).

In parallel with these morphological shifts, epiphytic microbial colonization increased with Cd concentration. CK fronds displayed intact, lightly wrinkled surfaces with few, small-aperture stomata and sparse microbial presence (Figure 1b–d). At 1 μM Cd, frond margins developed shallow, strip-like grooves and enlarged stomatal apertures, accompanied by modest microbial attachment (Figure 1f–h). Under 10 μM Cd, deeper surface grooves, localized structural damage, and slight edge wrinkling were evident; stomata remained abundant but frequently showed adhesive occlusions within the apertures, and a dense bacterial layer covered the frond surface (Figure 1j–l). Together, these observations indicate that Cd stress simultaneously remodels stomatal architecture and promotes epiphytic microbial enrichment on *S. polyrhiza* fronds.

### 3.2. Microbiome Profiling of S. polyrhiza Fronds Under Cadmium Stress

**Figure 2:**
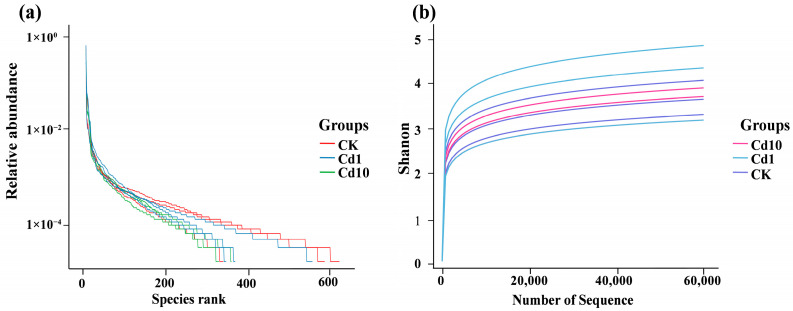
Diversity curves for *S. polyrhiza* frond–associated bacterial communities. (**a**) Species rank-abundance curve. (**b**) Species rarefaction curve. CK, 0 μM Cd; Cd1, 1 μM Cd; Cd10, 10 μM Cd.

To elucidate how cadmium stress influences microbial colonization on *S. polyrhiza* fronds, we performed high-throughput 16S rRNA gene sequencing of bacteria associated with frond surfaces following exposure to 0, 1, and 10 μM Cd. Across all treatments, a total of 26 phyla, 64 classes, 125 orders, 206 families, 406 genera, and 514 species were detected. The rank-abundance curves (Figure 2a) illustrate both species richness and evenness: a longer horizontal span indicates higher species abundance, while a smoother curve reflects greater community uniformity. In our data, each treatment’s curve extends widely before plateauing, confirming that sequencing captured a rich and balanced community. Rarefaction (dilution) curves (Figure 2b) further demonstrate that as sequencing depth increased, the observed number of species rose and then reached a stable asymptote, indicating that our sampling effort was sufficient to survey the majority of bacterial diversity present.

### 3.3. Alpha Diversity of the Epiphytic Bacterial Community on S. polyrhiza Fronds

**Figure 3:**
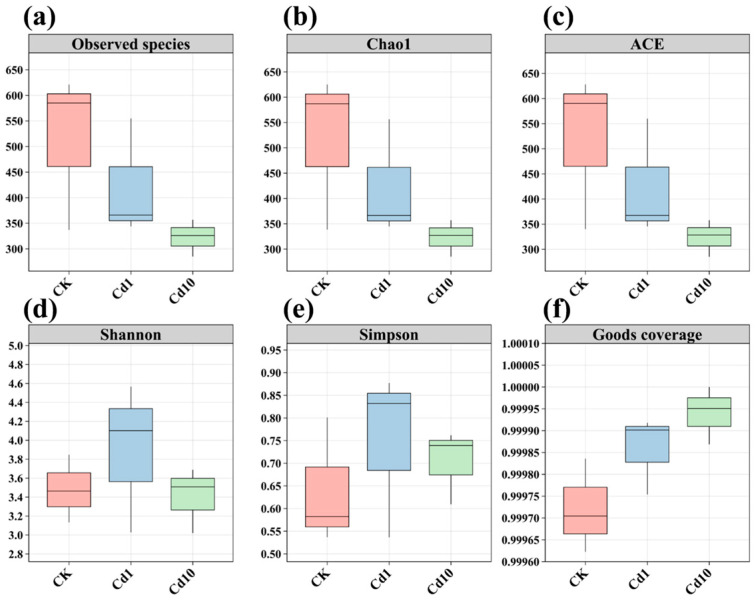
Alpha diversity indices of bacterial communities in *S. polyrhiza* fronds under different Cd treatments. (**a**) Observed species, (**b**) Chao1, (**c**) ACE, (**d**) Shannon, (**e**) Simpson, (**f**) Goods coverage. CK, 0 μM Cd; Cd1, 1 μM Cd; Cd10, 10 μM Cd.

Alpha diversity metrics were employed to assess the richness and evenness of frond-associated bacterial communities (Figure 3, Table S1). Community richness, as indicated by observed species counts, Chao1, and ACE indices, was highest in the control (CK), intermediate in 1 μM Cd (Cd1), and lowest in 10 μM Cd (Cd10) treatments. Both observed species and Chao1 values declined significantly in Cd1 and Cd10 compared to CK. In contrast, diversity metrics (Shannon and Simpson indices) peaked under Cd1, even surpassing the control, suggesting that moderate Cd stress fosters a more even community structure. Collectively, Cd exposure reduced overall bacterial richness on *S. polyrhiza* fronds while enhancing community evenness, indicative of a shift toward a more balanced microbiome.

### 3.4. Beta Diversity Analysis of Bacterial Communities in S. polyrhiza Fronds

**Figure 4:**
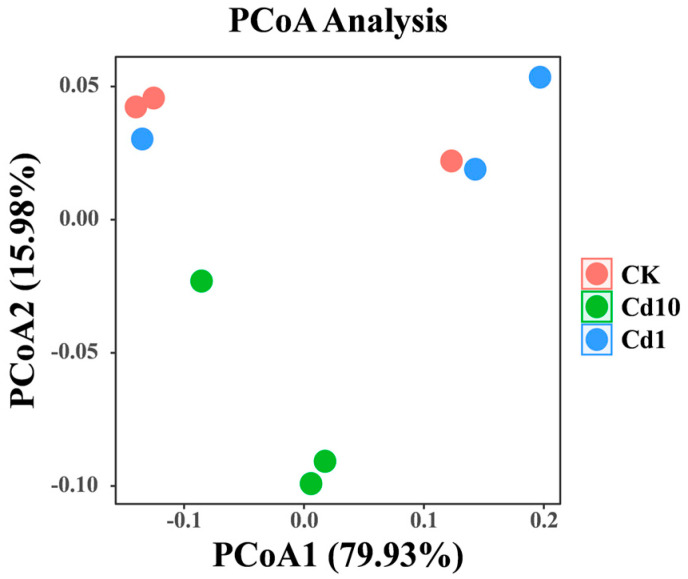
Principal coordinate analysis (PCoA) of frond bacterial communities in *S. polyrhiza* under varying Cd concentrations. CK, 0 μM Cd; Cd1, 1 μM Cd; Cd10, 10 μM Cd.

The differences in the species diversity of the microbial communities among the different samples are shown in Figure 4. Principal coordinate analysis (PCoA) was used to evaluate the similarities and differences in microbial community structure between the sample groups. The figure shows that the PCoA1 and PCoA2 axes explained 79.93% and 15.98% of the variation in the bacterial community composition, respectively. In particular, the points of the Cd10 treatment group and the control group had better separation (Figure 4), indicating that the untreated *S. polyrhiza* fronds differed between the international flora and the 10 μM cadmium treatment. Compared with the control treatment, 10 μM Cd stress changed the structure of the *S. polyrhiza* bacterial community.

### 3.5. Microbial Community Structure and Composition Under Cd Treatment

**Figure 5:**
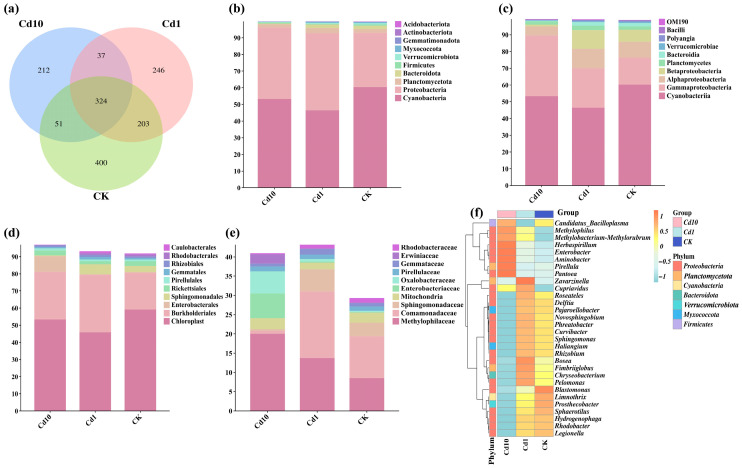
Taxonomic composition of *S. polyrhiza* frond microbiomes under Cd stress. (**a**) Venn diagram showing unique and shared ASVs among treatments. (**b**–**e**) Relative abundance of the top 10 taxa at the phylum (**b**), class (**c**), order (**d**), and family (**e**) levels. (**f**) Heatmap of the top 30 genera; color scale indicates relative abundance from low (deep red) to high (deep blue). Cd10, 10 μM Cd; Cd1, 1 μM Cd; CK, 0 μM Cd.

To assess how increasing Cd concentrations reshape *S. polyrhiza* frond–associated microbiomes, we first processed 16S rRNA gene reads through DADA2, yielding 1473 high-quality ASVs (Table S2). Venn analysis (Figure 5a) revealed 400, 246, and 212 unique ASVs in the CK, Cd1, and Cd10 groups, respectively, alongside 324 ASVs shared across all treatments, indicating both a core microbiome and treatment-specific taxa.

At the phylum level (Figure 5b), Cyanobacteria dominated in CK but declined by 23% under Cd1 (to 46.51%) and remained low in Cd10. Proteobacteria increased markedly under Cd stress: from 32.6% in CK to 46.26% in Cd1 (+42%) and 42.68% in Cd10 (+31%). Bacteroidota, the fourth most abundant phylum, decreased significantly in Cd10 compared to CK (*p* < 0.05). Within Proteobacteria, class-level (Figure 5c) changes were pronounced: Cyanobacteriia averaged 53.4% across all samples, followed by Gammaproteobacteria (25.2%), Alphaproteobacteria (8.8%), and Betaproteobacteria (6.5%). Notably, Gammaproteobacteria rose to 36.2% in Cd10—1.3-fold higher than CK (15.96%) (*p* < 0.05).

At the order level (Figure 5d), Chloroplast and Burkholderiales remained abundant across treatments. Cd10 uniquely enriched Enterobacterales (*p* < 0.05) while significantly reducing Sphingomonadales (*p* < 0.05). Examining the family level (Figure 5e), Methylophilaceae was most prevalent overall, exhibiting a clear “Cd10 > Cd1 > CK” trend. Comamonadaceae and Sphingomonadaceae ranked second and third, respectively. Finally, the genus-level heatmap (Figure 5f) of the top 30 genera highlights a Cd-dependent shift in community composition. In Cd10, *Methylophilus* peaked at 20.03% of total reads (versus 8.44% in CK), followed by Cd1 (13.75%). Genera known for metal resistance and detoxification—*Herbaspirillum*, *Enterobacter*, *Pantoea*, *Rhodobacter*, and *Hydrogenophaga*—also increased significantly with rising Cd concentrations (*p* < 0.05). These compositional shifts confirm that *S. polyrhiza* frond microbiota not only tolerate but actively respond to cadmium stress through selective enrichment of specific taxa.

### 3.6. Linear Discriminant Analysis Effect Size (LEfSe) for Identification of Differential Bacterial Community in S. polyrhiza Fronds

**Figure 6:**
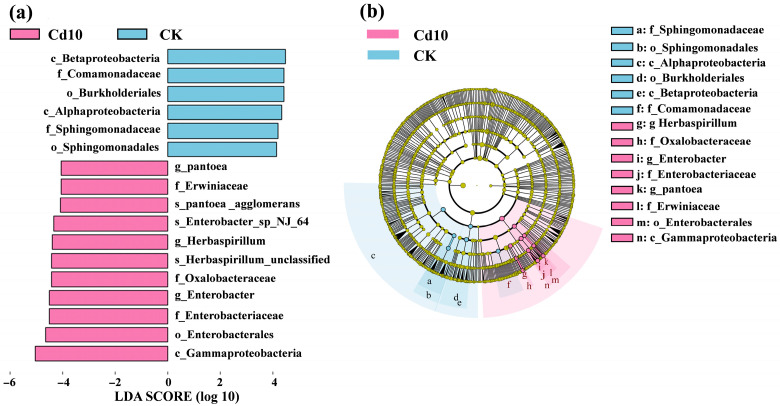
LEfSe analysis of differential taxa between Cd10 and CK groups. (**a**) LDA scores (>4) identifying discriminative taxa from class to species (*p* < 0.05). (**b**) Cladogram illustrating enriched taxa (inner circles: higher ranks; outer circles: genera). Cd10, 10 μM Cd; CK, 0 μM Cd.

Using an LDA score threshold of 4, LEfSe identified taxa that discriminated between CK and Cd10 treatments across multiple taxonomic levels (class, order, family, genus, and species) (Figure 6). Following treatment with 10 μM Cd, the relative abundances of Gammaproteobacteria, Enterobacterales, Enterobacteriaceae, Erwiniaceae, Oxalobacteraceae, *Pantoea*, *Enterobacter*, and *Herbaspirillum* in *S. polyrhiza* frond were significantly higher than those in the control group. In the CK group, the following taxa were significantly enriched: Alphaproteobacteria, Betaproteobacteria, Sphingomonadaceae, Sphingomonadales, Comamonadaceae, and Burkholderiales.

### 3.7. Functional Prediction of the Epiphytic Bacteria Community on S. polyrhiza Fronds

**Figure 7:**
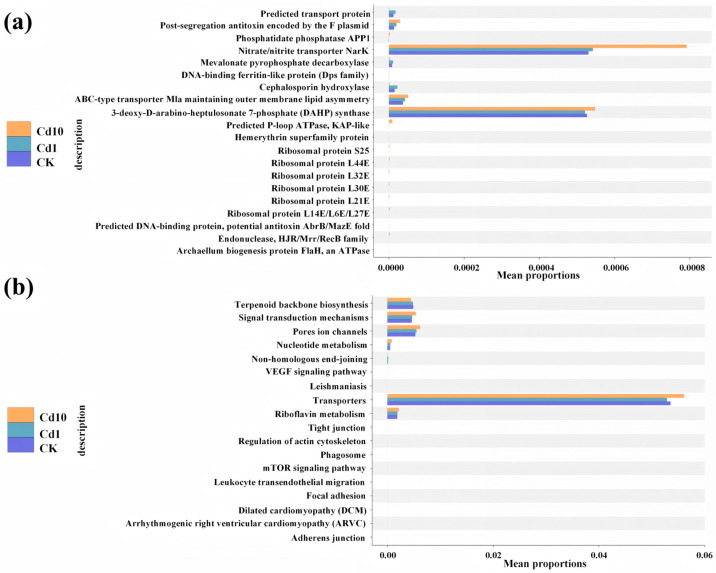
Predicted functional profiles of bacterial communities in *S. polyrhiza* fronds under Cd stress. (**a**) Enrichment of COG functional categories. (**b**) Significantly enriched KEGG pathways between treatments. Cd10, 10 μM Cd; Cd1, 1 μM Cd; CK, 0 μM Cd.

To infer the metabolic potential of frond-associated bacterial communities under Cd stress, we applied PICRUSt2 to 16S rRNA gene data, mapping predicted gene families to COG and KEGG pathways (Figure 7). The COG database classifies orthologous gene products by comparing protein sequences across diverse taxa. In Cd10 samples, several GO-classified functions—such as predicted transport proteins, mevalonate pyrophosphate decarboxylase, and cephalosporin hydroxylase—were significantly depleted relative to CK (*p* < 0.05). Conversely, Cd exposure enriched the abundance of genes encoding the F-plasmid postsegregation antitoxin, the nitrate/nitrite transporter NarK, and components of the ABC-type Mla transport system (including the STAS-domain–containing MlaB), which maintains outer membrane lipid asymmetry (*p* < 0.05).

The KEGG analysis systematically maps predicted genes to cellular pathways. Both Cd1 and Cd10 treatments increased the relative abundance of pathways involved in signal transduction and ion channel function, as well as nucleotide and riboflavin metabolism, compared to CK (*p* < 0.05). In contrast, the terpenoid backbone biosynthesis pathway was significantly downregulated under Cd stress (*p* < 0.05). Notably, the abundance of general transporter pathways in Cd10 exceeded that in CK, whereas Cd1 exhibited reduced transporter pathway representation. These functional shifts suggest that *S. polyrhiz*-associated bacteria may remodel their metabolic repertoire—particularly transport, signaling, and detoxification functions—to adapt to cadmium stress.

## 4. Discussion

### 4.1. Effects of Cadmium Stress on S. polyrhiza Leaf Physiology

An intriguing observation from our SEM analysis was that Cd treatment dramatically altered stomatal traits: compared with the control group, the maximum increases in stomatal width and stomatal area in the treatment groups were 1.7-fold and 1.46-fold, respectively, while the maximum increase in stomatal density in the treatment groups was 4.21-fold (Table 1; Figure 1). Stomata are the main channels for gas exchange and transpiration between plant leaves and the surrounding atmosphere and serve as direct entrances for heavy metal absorption [40,41]. Stomata adapt to environmental changes (carbon dioxide concentration, humidity and temperature) by changing their pore size [42,43]. The stomatal opening size affects the photosynthesis rate, transpiration rate and nutrient uptake efficiency of plants [44,45]. Cadmium exposure is associated with morphological abnormalities in plant leaves and stomatal development—for instance, studies on pea (*Pisum sativum*) have shown that impaired stomatal development and reduced gas exchange efficiency under increasing Cd, alongside broader morphological and physiological deficits across tissues [46]. In eggplant cultivars, Cd stress induces physiological changes in which modulation of stomatal density is a key determinant of differential Cd translocation to shoots [47]. Most studies have shown that heavy metals can reduce or close the stomata of plant leaves. For example, studies have shown that, compared with those of the blank control group, the leaf edges of duckweed treated with 2 mg/L Cd are damaged, the surface is rough, and the stomata are closed; moreover, Cd induces stomatal closure to reduce water loss [19]. Studies have shown that soil Ni contamination can significantly reduce the stomatal area of corn leaves. This is because Ni toxicity reduces the relative leaf water content (RWC), forcing stomata to shrink to reduce water evaporation [48]. Our study extends these observations to *S. polyrhiza*, showing that moderate Cd stress (1 μM) disrupts normal stomatal regulation—likely reflecting Cd-induced physiological and cellular dysfunction—while higher stress (10 μM) maintains stomatal openness, perhaps due to leaf-surface microbes stabilizing Cd, sequestering free ions, and lessening tissue damage [49,50]. Meanwhile, cadmium induces the formation of more furrows on frond surfaces, and the grooves and stomata in fronds serve as preferential attachment sites for bacteria [51]. Studies have shown that microorganisms can alter the morphology and density of stomata on plant leaves, as well as regulate stomatal opening and closing behaviors [52]. Certain microorganisms can alleviate cadmium-induced damage to plants by enhancing the plant antioxidant systems and regulating nutrient uptake, thereby mitigating the negative impacts on stomatal function [53]. Some microorganisms can also produce enzymes and metabolites to enhance plant tolerance related to heavy metals, maintaining relatively normal stomatal function [54]. These microbe–stomata interactions warrant deeper investigation. Future studies that target stomatal responses may thus yield critical insights into *S. polyrhiza*’s Cd-accumulation mechanisms.

### 4.2. Effects of Cadmium Stress on the Epiphytic Bacterial Composition of S. polyrhiza Fronds

Characterizing the frond microbiome of *S. polyrhiza* across a Cd gradient clarifies the ecological relevance of microbial indicators and reveals how leaf–microbe interactions jointly mediate Cd responses. In our dataset, Cd exposure altered community richness and diversity—reflected by observed species, Chao1, ACE, Shannon, and Simpson indices—underscoring the sensitivity of the epiphytic microbiome to metal stress. Microbial diversity is fundamental to plant health [55,56], with intra-community interactions forming protective barriers against pathogens and heavy-metal damage [57]. At the phylum level, Proteobacteria dominated under Cd treatment, consistent with their recognized metal tolerance. Members of this phylum can exploit toxic substrates and frequently harbor oxidase and other resistance genes that support heavy-metal transformation and detoxification, thereby offering prospects for phytoremediation [58,59,60]. At finer taxonomic resolution, the genera *Herbaspirillum*, *Enterobacter*, and *Pantoea* increased with Cd concentration, suggesting key roles in abiotic-stress mitigation. *Herbaspirillum* engages in beneficial plant associations, with glycoconjugates and lipopolysaccharides implicated in metal adsorption and immobilization [61,62]; in consortia, it can enhance soil enzyme activities, stimulate root growth, activate antioxidant defenses, and reduce Pb/Cd bioavailability and shoot translocation [63]. *Enterobacter* exhibits Cd tolerance, bioaccumulation capacity, and growth-promoting traits linked to enhanced central metabolism (pyruvate/TCA) and oxidative-stress proteins [64,65]. *Pantoea* often dominates leaf surfaces under metal stress—reaching high abundance in rice leaves—with biosorption, bioprecipitation, and exopolysaccharide secretion contributing to stress alleviation [66]. Collectively, the enrichment of these metal-tolerant taxa under Cd stress likely facilitates detoxification, supports host growth, and improves Cd resilience [67]. Meanwhile, results from PCoA and LDA indicated that Cd stress induced differentiation in the bacterial communities associated with the fronds of *S. polyrhiza*. This suggests that exposure to different concentrations of the heavy metal Cd enables the formation of representative microbial communities on *S. polyrhiza* fronds, which may facilitate the survival and proliferation of microbial taxa that synergize with cadmium. To cope with the toxic effects of cadmium, the microbiota of *S. polyrhiza* undergoes structural restructuring, and this restructuring serves as the foundation for the subsequent functional differentiation of the microbiota. Targeted studies of these genera—spanning mechanistic genetics, metabolite profiling, and synthetic-community reconstruction—will be pivotal for deciphering bacterial assistance in plant Cd tolerance and for guiding plant–microbe remediation strategies across species.

### 4.3. Functional Differentiation of S. polyrhiza Frond Bacterial Communities Under Cadmium Stress

PICRUSt2-based functional inference (COG and KEGG) revealed that Cd exposure drove marked functional divergence in the leaf-associated microbiome of *S. polyrhiza*. Relative abundances of genes linked to the nitrate/nitrite transporter NarK, the ABC-type Mla transport system (which maintains outer-membrane lipid asymmetry), signal transduction mechanisms, and pore/ion channels were significantly elevated under Cd treatment (*p* < 0.05; Figure 7). These shifts indicate that Cd stress reprograms microbial metabolic potential and resource allocation, favoring functions associated with transport, signaling, and membrane homeostasis [68]. Increases in these functional categories suggest that constituent bacteria possess enhanced Cd tolerance and can sustain core metabolism on frond surfaces during metal stress. Consistent with this view, pathways related to the metabolism, biosynthesis, and degradation of amino acids, fatty acids, and nucleotides often rise in Cd-contaminated systems, improving microbial resilience [69]. Species and specific strains with higher expression of ABC transporters accumulate less metals [70]. Studies have shown that YCF1 from *Saccharomyces cerevisiae*, which belongs to the ATP-binding cassette (ABC) transporter family, uses energy derived from ATP hydrolysis to pump cadmium ions bound to glutathione (GSH) in the cytoplasm into vacuoles for sequestration. This process reduces the toxicity of free cadmium in the cytoplasm, thereby enabling cadmium detoxification in yeast [71]. In parallel, studies in crops highlight energy metabolism, antioxidant defense, metal transport, and ion homeostasis as key determinants of Cd tolerance [72]. At the community scale, Cd can restructure rhizosphere functional profiles—impacting broad categories such as “Metabolism,” “Genetic Information Processing,” and “Organismal Systems”—with potential consequences for Cd bioavailability and plant–microbe interactions [73]. These functional adaptive changes are common adaptive strategies of microbial communities under environmental stress, which are aimed at ensuring the maintenance of core functions necessary for survival as well as synergistic interactions with the host. Taken together, our results support a model in which prolonged Cd exposure selects for frond-associated bacteria equipped with transport- and signaling-intensive strategies, enabling stable membrane function, metal handling, and metabolic continuity. Future work should isolate and functionally validate these candidate taxa and pathways—via metagenomics/metatranscriptomics and synthetic community assays—to assess their bioremediation potential in Cd-contaminated environments.

## 5. Conclusions

In our study, cadmium exposure to the leaves of *S. polyrhiza* induced concentration-dependent chlorosis and wilting symptoms. Concurrently, it significantly altered leaf stomatal structure and leaf surface morphology—with grooves forming on the frond surface. Cadmium stress modified the leaf-associated bacterial community structure, characterized by the enrichment of Proteobacteria and key genera (*Herbaspirillum*, *Enterobacter*, *Pantoea*). This may constitute one of the mechanisms underlying duckweed’s tolerance to cadmium. Meanwhile, the Cd-exposed microbiota remodeled the nitrite transporter NarK, signal transduction systems, and ion-channel systems to facilitate detoxification. This likely represents an adaptation of organisms to enhance stress resistance. For future research, these dominant bacterial strains could be specifically isolated. Using transcriptomics or metabolomics approaches, regulatory mechanisms can be investigated at the protein and gene levels to further explore the mechanisms of Cd tolerance and detoxification. Additionally, the synergistic effects of combined remediation by duckweed and microorganisms can be further studied to improve the efficiency of co-remediation of Cd contamination by plants and microorganisms.

## Figures and Tables

**Table 1:** Characteristic parameters of the stomata of *S. polyrhiza* fronds under different concentrations of cadmium.

Treatments	Stomatal Length (μm)	Stomatal Width (μm)	Stomatal Area (μm^2^)	Stomatal Density (No./mm^2^)
CK	12.25 ± 1.29	1.25 ± 0.39 b	12.12 ± 3.96 b	36.28 ± 12.32 b
Cd1	12.43 ± 1.41	3.38 ± 1.48 a	29.81 ± 13.04 a	156 ± 21.62 a
Cd10	12.39 ± 0.7	2.53 ± 0.61 a	24.74 ± 4.16 a	189.11 ± 27.94 a

CK, 0 μM Cd; Cd1, 1 μM Cd; Cd10, 10 μM Cd. Data in the table are presented as mean ± standard deviation. Different lowercase letters within a column denote significant differences among samples (*p* < 0.05).

## Data Availability

The original contributions presented in this study are included in the article/Supplementary Materials. Further inquiries can be directed to the corresponding author(s).

## Supplementary Materials

The following supporting information can be downloaded at: https://www.mdpi.com/article/10.3390/microorganisms13112423/s1, Table S1: Microbial diversity indices of giant duckweed (*Spirodela polyrhiza*) fronds; Table S2: ASV table for giant duckweed (*Spirodela polyrhiza*) fronds with taxonomy.

## References

[1] Khan M.A., Wani G.A., Majid H., Farooq F.U., Reshi Z.A., Husaini A.M., Shah M.A. (2020). Differential Bioaccumulation of Select Heavy Metals from Wastewater by *Lemna minor*. Bull. Environ. Contam. Toxicol..

[2] Xu H., Yu C., Xia X., Li M., Li H., Wang Y., Wang S., Wang C., Ma Y., Zhou G. (2018). Comparative Transcriptome Analysis of Duckweed (*Landoltia punctata*) in Response to Cadmium Provides Insights into Molecular Mechanisms Underlying Hyperaccumulation. Chemosphere.

[3] Li L., Wang L., Chen K., Ming R., Yang Y., Zhang Y., Lu P. (2025). Exploring the Effects of Environmentally Relevant Concentrations of Buprofezin and Cadmium on Tadpoles: A Phenotypic and Molecular Analysis. Environ. Res..

[4] Sattar S., Yahya M., Aslam S., Hussain R., Shah S.M.M., Rauf Z., Zamir A., Ullah R., Shahzad A. (2025). Environmental Occurrence, Hazards, and Remediation Strategies for the Removal of Cadmium from the Polluted Environment. Results Eng..

[5] Suwazono Y., Kido T., Nakagawa H., Nishijo M., Honda R., Kobayashi E., Dochi M., Nogawa K. (2009). Biological Half-Life of Cadmium in the Urine of Inhabitants after Cessation of Cadmium Exposure. Biomarkers.

[6] Luo Y., Huang X., Sha A., He J., Chen X., Xiao W., Peng L., Zou L., Liu B., Li Q. (2025). Analysis of Growth Physiological Changes and Metabolome of Highland Barley Seedlings under Cadmium (II) Stress. Environ. Pollut..

[7] Haider F.U., Liqun C., Coulter J.A., Cheema S.A., Wu J., Zhang R., Wenjun M., Farooq M. (2021). Cadmium Toxicity in Plants: Impacts and Remediation Strategies. Ecotoxicol. Environ. Saf..

[8] Zhu T., Li L., Duan Q., Liu X., Chen M. (2021). Progress in Our Understanding of Plant Responses to the Stress of Heavy Metal Cadmium. Plant Signal. Behav..

[9] Mushtaq G., Agrawal S., Kushwah A., Kumar A., Lone R. (2025). Cadmium Toxicity in Plants and Its Remediation Management: A Review. Plant Stress.

[10] Hermans C., Chen J., Coppens F., Inzé D., Verbruggen N. (2011). Low Magnesium Status in Plants Enhances Tolerance to Cadmium Exposure. New Phytol..

[11] Bekkai D., Chiofalo M.T., Torre D., Mileto S., Genovese G., Cimino F., Toscano G., Iannazzo D., Trifilò P. (2024). Chronic Mild Cadmium Exposure Increases the Vulnerability of Tomato Plants to Dehydration. Plant Physiol. Biochem..

[12] Mahajan P., Kaushal J. (2018). Role of Phytoremediation in Reducing Cadmium Toxicity in Soil and Water. J. Toxicol..

[13] Castañeda C.R., García-Martínez B., Zamudio-Cuevas Y., Fernández-Torres J., Martínez-Flores K. (2025). Cadmium Exposure and Its Role in Joint Disease: A Brief Review of Experimental and Population-Based Evidence. J. Trace Elem. Med. Biol..

[14] Nehzomi Z.S., Shirani K. (2025). The Gut Microbiota: A Key Player in Cadmium Toxicity—Implications for Disease, Interventions, and Combined Toxicant Exposures. J. Trace Elem. Med. Biol..

[15] Zhao Z., Shi H., Liu C., Kang X., Chen L., Liang X., Jin L. (2018). Duckweed Diversity Decreases Heavy Metal Toxicity by Altering the Metabolic Function of Associated Microbial Communities. Chemosphere.

[16] Liu Q., Liu S., Wang D., Sun D., Ge Y., Zhang S., Li G., Jho E.H., Joo J.C., Zhao X. (2025). Decoupling Soil pH and Geography: Universal Drivers of Cadmium Bioavailability in Rice across Terrains. J. Environ. Manag..

[17] Sharma S., Kumar T., Das D.K., Mittal A., Verma N. (2025). Vinod Phytoremediation of Heavy Metals in Soil—Concepts, Advancements, and Future Directions. J. Soil Sci. Plant Nutr..

[18] Deng S., Zhang X., Zhu Y., Zhuo R. (2024). Recent Advances in Phyto-Combined Remediation of Heavy Metal Pollution in Soil. Biotechnol. Adv..

[19] Wang X., Hu L., Wu D., Huang T., Zhang B., Cai G., Gao G., Liu Z., Huang X., Zhong Z. (2022). Large-Scale Screening and Characterization of Cd Accumulation and Ultrastructural Deformation in Duckweed. Sci. Total Environ..

[20] Song L., Zhou J., Xu X., Na M., Xu S., Huang Y., Zhang J., Li X., Zheng X. (2024). Inoculation of Cadmium-Tolerant Bacteria to Regulate Microbial Activity and Key Bacterial Population in Cadmium-Contaminated Soils during Bioremediation. Ecotoxicol. Environ. Saf..

[21] Genchi G., Sinicropi M.S., Lauria G., Carocci A., Catalano A. (2020). The Effects of Cadmium Toxicity. Int. J. Environ. Res. Public Health.

[22] Cozma P., Roșca M., Minuț M., Gavrilescu M. (2025). Phytoremediation: A Sustainable and Promising Bio-Based Approach to Heavy Metal Pollution Management. Sci. Total Environ..

[23] Baek G., Saeed M., Choi H.-K. (2021). Duckweeds: Their Utilization, Metabolites and Cultivation. Appl. Biol. Chem..

[24] Chen S., Xu J., Peng L., Cheng Z., Kuang X., Li D., Peng C., Song H. (2023). Cadmium Accumulation in Rice Grains Is Mitigated by Duckweed-like Hydrophyte through Adsorption and Increased Ammonia Nitrogen. Sci. Total Environ..

[25] Sharma R., Lenaghan S.C. (2022). Duckweed: A Potential Phytosensor for Heavy Metals. Plant Cell Rep..

[26] Xie Y., Zhao Y., Li Y., Wang Y., Pei J., Xu H. (2024). Cadmium Induced Changes in Rhizosphere Microecology to Enhance Cd Intake by *Ligusticum sinense* cv. Chuanxiong. J. Hazard. Mater..

[27] Wang M., Yu L., Wang J., Qin L., Sun X., Liu J., Han Y., Chen S. (2025). Chemotaxis of Rhizosphere *Pseudomonas* sp. Induced by Foliar Spraying of Lanthanum Reduces Cadmium Uptake by Pakchoi. J. Hazard. Mater..

[28] Yang J., Zhao X., Wang X., Xia M., Ba S., Lim B.L., Hou H. (2024). Biomonitoring of Heavy Metals and Their Phytoremediation by Duckweeds: Advances and Prospects. Environ. Res..

[29] Niu S., Li T., Liu L., Bao X., Yang X., Song H., Li Y., Bai J., He L., Wang Q. (2025). Mechanistic Study on the Mitigation of Cadmium Accumulation in *Ligusticum sinense* cv. Chuanxiong Through Plant Growth-Promoting Rhizobacteria *Arthrobacter* sp. CX-2. Plant Stress..

[30] Chi Y., Ma X., Chu S., You Y., Chen X., Wang J., Wang R., Zhang X., Zhang D., Zhao T. (2025). Nitrogen Cycle Induced by Plant Growth-Promoting Rhizobacteria Drives “Microbial Partners” to Enhance Cadmium Phytoremediation. Microbiome.

[31] Yang X., Tan A.-J., Zheng M.-M., Feng D., Mao K., Yang G.-L. (2023). Physiological Response, Microbial Diversity Characterization, and Endophytic Bacteria Isolation of Duckweed under Cadmium Stress. Sci. Total Environ..

[32] Parihar A., Malaviya P. (2023). Textile Wastewater Phytoremediation Using *Spirodela polyrhiza* (L.) Schleid. Assisted by Novel Bacterial Consortium in a Two-Step Remediation System. Environ. Res..

[33] Manzoor M., Guan D.-X., Ma L.Q. (2025). Plant-Microbiome Interactions for Enhanced Crop Production under Cadmium Stress: A Review. Sci. Total Environ..

[34] Adams W., Blust R., Dwyer R., Mount D., Nordheim E., Rodriguez P.H., Spry D. (2020). Bioavailability Assessment of Metals in Freshwater Environments: A Historical Review. Environ. Toxicol. Chem..

[35] Logue J.B., Stedmon C.A., Kellerman A.M., Nielsen N.J., Andersson A.F., Laudon H., Lindström E.S., Kritzberg E.S. (2016). Experimental Insights into the Importance of Aquatic Bacterial Community Composition to the Degradation of Dissolved Organic Matter. ISME J..

[36] Callahan B.J., McMurdie P.J., Rosen M.J., Han A.W., Johnson A.J.A., Holmes S.P. (2016). DADA2: High-Resolution Sample Inference from Illumina Amplicon Data. Nat. Methods.

[37] Bolyen E., Rideout J.R., Dillon M.R., Bokulich N.A., Abnet C.C., Al-Ghalith G.A., Alexander H., Alm E.J., Arumugam M., Asnicar F. (2019). Reproducible, Interactive, Scalable and Extensible Microbiome Data Science Using QIIME 2. Nat. Biotechnol..

[38] Douglas G.M., Maffei V.J., Zaneveld J.R., Yurgel S.N., Brown J.R., Taylor C.M., Huttenhower C., Langille M.G.I. (2020). PICRUSt2 for Prediction of Metagenome Functions. Nat. Biotechnol..

[39] Drost W., Matzke M., Backhaus T. (2007). Heavy Metal Toxicity to Lemna Minor: Studies on the Time Dependence of Growth Inhibition and the Recovery after Exposure. Chemosphere.

[40] Ouyang X., Ma J., Liu Y., Li P., Wei R., Chen Q., Weng L., Chen Y., Li Y. (2023). Foliar Cadmium Uptake, Transfer, and Redistribution in Chili: A Comparison of Foliar and Root Uptake, Metabolomic, and Contribution. J. Hazard. Mater..

[41] Zhu Z., Peng J., Yu P., Fei J., Huang Z., Deng Y., Yang X., Luo J., Li T., Huang Y. (2025). Foliar Uptake, Translocation and Its Contribution to Cadmium Accumulation in Rice. Sci. Total Environ..

[42] Kinoshita T., Toh S., Torii K.U. (2021). Chemical Control of Stomatal Function and Development. Curr. Opin. Plant Biol..

[43] Qi X., Torii K.U. (2018). Hormonal and Environmental Signals Guiding Stomatal Development. BMC Biol..

[44] Shao X., Yu P., Zuo M., Tong Z., Huang Z., Xie Z., Chang R., Peng J., Deng Y., Huang Y. (2025). Screening of Rice Varieties with Low Accumulation of Heavy Metals Based on Leaf Morphology. J. Plant Physiol..

[45] Wang Y., Wang Y., Tang Y., Zhu X.-G. (2022). Stomata Conductance as a Goalkeeper for Increased Photosynthetic Efficiency. Curr. Opin. Plant Biol..

[46] Almuwayhi M.A. (2021). Effect of Cadmium on the Molecular and Morpho-Physiological Traits of *Pisum sativum* L. Biotechnol. Biotechnol. Equip..

[47] Shen C., Huang Y.-Y., Liao Q., Huang B.-F., Xin J.-L., Wang L., Fu H.-L. (2023). Characterization of Cadmium Accumulation Mechanism between Eggplant (*Solanum melongena* L.) Cultivars. Front. Plant Sci..

[48] Bijanzadeh E., Boostani H.R., Hardie A.G., Najafi-Ghiri M. (2024). Co-Application of Silicon and Biochar Affected Anatomical and Biochemical Properties of Corn Leaf (*Zea mays* L.) Under Soil Nickel Toxicity. Heliyon.

[49] Wang X., Cai D., Ji M., Chen Z., Yao L., Han H. (2022). Isolation of Heavy Metal-Immobilizing and Plant Growth-Promoting Bacteria and Their Potential in Reducing Cd and Pb Uptake in Water Spinach. Sci. Total Environ..

[50] Gupta P., Bhatnagar A.K. (2015). Spatial Distribution of Arsenic in Different Leaf Tissues and Its Effect on Structure and Development of Stomata and Trichomes in Mung Bean, *Vigna radiata* (L.) Wilczek. Environ. Exp. Bot..

[51] Yang H., Liu J., Ma M., Tan Z., Zhang K., Sun R., Zhan X., Cui D. (2025). Leaf Development and Its Interaction with Phyllospheric Microorganisms: Impacts on Plant Stress Responses. Plant Stress.

[52] Llorente B.E., Alasia M.A., Larraburu E.E. (2016). Biofertilization with *Azospirillum brasilense* Improves In Vitro Culture of *Handroanthus ochraceus*, a Forestry, Ornamental and Medicinal Plant. New Biotechnol..

[53] Khanna K., Kohli S.K., Ohri P., Bhardwaj R., Ahmad P. (2022). Agroecotoxicological Aspect of Cd in Soil–Plant System: Uptake, Translocation and Amelioration Strategies. Environ. Sci. Pollut. Res..

[54] Boyno G. (2025). Biological Defence against Cadmium Stress in Wheat with Arbuscular Mycorrhizal Fungi and Trichoderma: Synergistic Effects on Plant and Soil Health. Plant Physiol. Biochem..

[55] Zhou X., Zhang Q., Yan Y., Qu J., Zhou J., Zhao J., Zhang J., Cai Z., Dai C., Huang X. (2025). Effects of Soil Management Strategies Based on Different Principles on Soil Microbial Communities and the Outcomes for Plant Health. Biol. Control.

[56] Trivedi P., Leach J.E., Tringe S.G., Sa T., Singh B.K. (2020). Plant–Microbiome Interactions: From Community Assembly to Plant Health. Nat. Rev. Microbiol..

[57] Yu F., He Z., Xin X., Shi X., Chen L., He X., Huang Y., Li Y. (2024). Evidence That Beneficial Microbial Inoculation Enhances Heavy Metal-Contaminated Soil Remediation: Variations in Plant Endophyte Communities. J. Hazard. Mater..

[58] Drzewiecka D. (2016). Significance and Roles of *Proteus* spp. Bacteria in Natural Environments. Microb. Ecol..

[59] Jarosławiecka A.K., Piotrowska-Seget Z. (2022). The Effect of Heavy Metals on Microbial Communities in Industrial Soil in the Area of Piekary Śląskie and Bukowno (Poland). Microbiol. Res..

[60] Jiang Y., Hu T., Peng O., Chen A., Tie B., Shao J. (2022). Impact of Heavy Metal Passivators on the Nitrogenase Activity and Diazotrophic Community in a Cadmium-Contaminated Paddy Field. Int. Biodeterior. Biodegrad..

[61] Venkatachalam J., Mohan H., Seralathan K.-K. (2023). Significance of *Herbaspirillum* sp. in Biodegradation and Biodetoxification of Herbicides, Pesticides, Hydrocarbons and Heavy Metals—A Review. Environ. Res..

[62] Velichko N.S., Grinev V.S., Fedonenko Y.P. (2020). Characterization of Biopolymers Produced by Planktonic and Biofilm Cells of *Herbaspirillum lusitanum* P6-12. J. Appl. Microbiol..

[63] Zhu X., Ju W., Beiyuan J., Chao H., Zhang Z., Chen L., Cui Q., Qiu T., Zhang W., Huang M. (2024). Bacterial Consortium Amendment Effectively Reduces Pb/Cd Bioavailability in Soil and Their Accumulation in Wheat. J. Environ. Manag..

[64] Ghosh A., Pramanik K., Bhattacharya S., Mondal S., Ghosh S.K., Maiti T.K. (2022). A Potent Cadmium Bioaccumulating *Enterobacter Cloacae* Strain Displays Phytobeneficial Property in Cd-Exposed Rice Seedlings. Curr. Res. Microb. Sci..

[65] Li Y., Shi X., Chen Y., Luo S., Qin Z., Chen S., Wu Y., Yu F. (2023). Quantitative Proteomic Analysis of the Mechanism of Cd Toxicity in *Enterobacter* sp. FM-1: Comparison of Different Growth Stages. Environ. Pollut..

[66] Gao H., Guo Z., He X., Yang J., Jiang L., Yang A., Xiao X., Xu R. (2024). Stress Mitigation Mechanism of Rice Leaf Microbiota amid Atmospheric Deposition of Heavy Metals. Chemosphere.

[67] Rana K.L., Kour D., Kaur T., Devi R., Yadav A.N., Yadav N., Dhaliwal H.S., Saxena A.K. (2020). Endophytic Microbes: Biodiversity, Plant Growth-Promoting Mechanisms and Potential Applications for Agricultural Sustainability. Antonie Leeuwenhoek.

[68] Wu Q., Lin X., Li S., Liang Z., Wang H., Tang T. (2023). Endophytic *Bacillus* sp. AP10 Harboured in Arabis Paniculata Mediates Plant Growth Promotion and Manganese Detoxification. Ecotoxicol. Environ. Saf..

[69] Feng G., Xie T., Wang X., Bai J., Tang L., Zhao H., Wei W., Wang M., Zhao Y. (2018). Metagenomic Analysis of Microbial Community and Function Involved in Cd-Contaminated Soil. BMC Microbiol..

[70] Jaskulak M., Grobelak A., Vandenbulcke F. (2020). Effects of Sewage Sludge Supplementation on Heavy Metal Accumulation and the Expression of ABC Transporters in *Sinapis alba* L. During Assisted Phytoremediation of Contaminated Sites. Ecotoxicol. Environ. Saf..

[71] Agnihotri A., Seth C.S. (2019). Transgenic Brassicaceae. Transgenic Plant Technology for Remediation of Toxic Metals and Metalloids.

[72] Ashraf H., Ghouri F., Sun L., Xia W., Ashraf S., Ashraf M.Z., Fu X., Ali S., Shahid M.Q. (2025). Energy Metabolism, Antioxidant Defense System, Metal Transport, and Ion Homeostasis Are Key Contributors to Cd Tolerance in SSSL Derived from Wild Rice. J. Hazard. Mater..

[73] Xu Y., Shen L., Chen M., Sun H., Fu L., Zhang G., Shen Q. (2025). Rhizosphere Microbial Communities of Bacteria and Fungi Responding to Cadmium Stress in Wheat. Crop Des..

